# The Application of Hyaluronic Acid/Alginate Sheet to Flexor Pollicis Longus Tendon Repair to Prevent Adhesion Formation: A Second Look

**DOI:** 10.7759/cureus.33147

**Published:** 2022-12-30

**Authors:** Emily R McDermott, Zachary Bowers, Julia A Nuelle

**Affiliations:** 1 Department of Orthopaedic Surgery, San Antonio Military Medical Center, San Antonio, USA; 2 Department of Orthopaedic Surgery, Missouri Orthopaedic Institute, Columbia, USA

**Keywords:** tendon repair, hyaluronic acid, flexor pollicis longus, flexor tendon, adhesion

## Abstract

Soft tissue traumas with tendon lacerations are challenging injuries to manage. Repair of these tendon injuries can be complicated by adhesions postoperatively, limiting patient function and satisfaction. Biologic agents have been developed to optimize tendon gliding after repair and can be used as adjuncts. When used in conjunction with a postoperative rehabilitation protocol, they can help restore function and range of motion (ROM). It is rare that the orthopedic surgeon can visualize a healed tendon repair as the results are often followed clinically. We present the case of a patient who underwent flexor tendon repair with the addition of hyaluronic acid (HA) and alginate tendon wrap. This same patient sustained a distal radius fracture one year later, allowing for a second look at the flexor tendon repair site during the operative fixation of his fracture.

## Introduction

Penetrating trauma to the hand and wrist is common; 10% of emergency department visits are hand injuries, of which 82% involve soft tissue [1]. Tendon lacerations can be a consequence of these types of injuries. One retrospective study found an incidence of 33.2 acute traumatic tendon injuries per 100,000 person-years [2,3]. While these injuries are relatively common, it is rare that the orthopedic surgeon is permitted the opportunity to reevaluate the tendon repair in an open fashion after the patient has healed. The results of tendon repair procedures are often followed clinically. We present the case of a patient who sustained an acute laceration of the right flexor pollicis longus (FPL) and underwent operative repair and placement of hyaluronic acid (HA) and alginate wrap over the tendon repair site. He subsequently underwent distal radius fracture operative fixation for a separate injury one year later, allowing direct visualization of the healed tendon repair.

## Case presentation

Initial procedure

**Figure 1:**
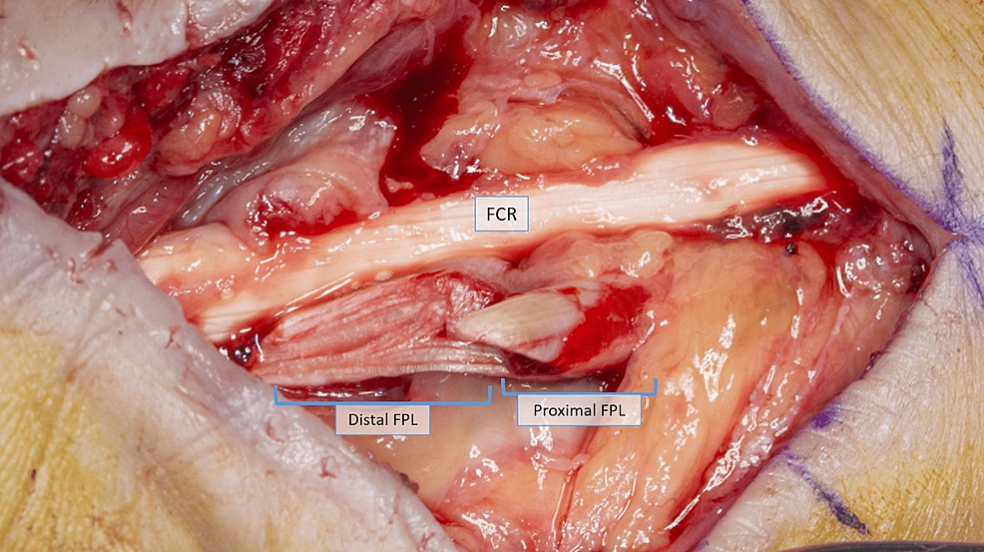
Day of the initial surgery: complete laceration of the FPL with a frayed distal edge with longitudinal splits FCR: flexor carpi radialis, FPL: flexor pollicis longus

The patient is a 68-year-old right-hand-dominant male who sustained an FPL tendon laceration when he fell on a glass, cutting his right wrist. The laceration crossed the distal wrist crease and extended from the distal forearm toward the thenar eminence. He was initially seen in the emergency department, where the wound was irrigated and closed, and he was discharged as he had an intact function and was otherwise neurovascularly intact. He again presented the next day with acute loss of the ability to flex his thumb. The patient was, at that time, noted to have an FPL laceration based on physical examination. He was taken to the operating room the same day and underwent irrigation and debridement and repair of the right FPL tendon laceration. The FPL was found to have a complex laceration in zone V with significant fraying of the distal end (Figure 1).

A modified Tsuge technique was performed using a looped 3-0 nylon suture. Given the irregular contour of the laceration and tendon ends, a braided 3-0 ethylene terephthalate suture was used to reinforce the repair in a running locking fashion. This technique resulted in a secure repair with no gapping. To limit unwanted postoperative adhesions, a HA/alginate sheet with activating solution (VersaWrap®, Alafair Biosciences, Austin, TX, USA) was then wrapped around the repair site.

The patient was splinted postoperatively. At his two-week postoperative appointment, he was able to actively flex the thumb interphalangeal joint (IPJ) and was transitioned to a thumb spica splint with the wrist at 20 degrees of extension, the metacarpophalangeal joints (MCPJs) at 15 degrees of flexion, and the IPJs at 30 degrees of flexion. He was instructed on passive range of motion (ROM) with occupational therapy. He progressed appropriately over the course of his postoperative visits. At his final follow-up appointment 10 weeks postoperatively, he was able to make a full composite fist and had 30 degrees of flexion in the right thumb IPJ when compared to 45 degrees on the contralateral extremity. He had resumed normal activity and was cleared without restrictions.

Second procedure

Approximately one year later, the patient sustained a right distal radius fracture after falling off a ladder. He underwent closed reduction and casting in the emergency department. He was indicated for operative fixation when repeat radiographs were taken one week later and demonstrated a loss of reduction.

**Figure 2:**
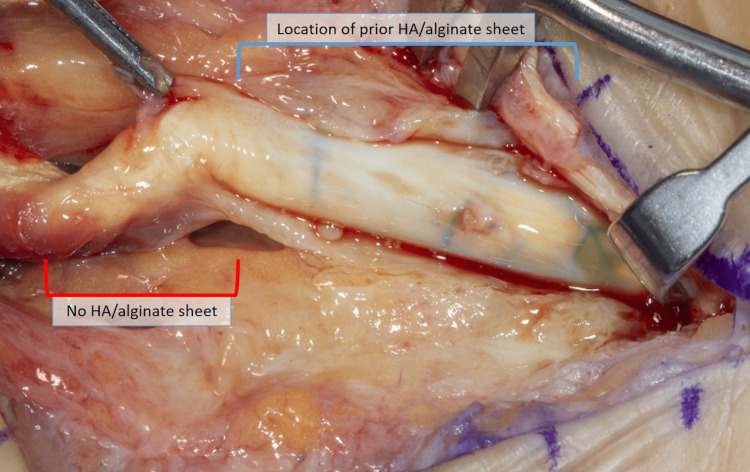
Day of the second surgery: a second look at the healed flexor pollicis longus tendon HA: hyaluronic acid

The patient subsequently underwent a right distal radius open reduction and internal fixation two days later. His original incision was incorporated into a modified Henry approach. The flexor carpi radialis (FCR) was identified and retracted, and the subsheath was incised. This exposure revealed the previously repaired FPL, which was intact. The repair was visualized and noted to have healed entirely. There were no adhesions in the area where the HA/alginate sheet had been applied. Proximal to the placement of the HA/alginate sheet, though, adhesions were noted (Figure 2). The tendon was carefully retracted, and the patient’s distal radius open reduction and internal fixation was performed without complication.

At the patient’s first postoperative visit two weeks following the second procedure, the incision was well-healed, and his sutures were removed. He was transitioned to a removable wrist brace at this time. He progressed appropriately without complication. At final clearance, he was again able to make a well-aligned composite fist with an intact range of motion and full flexion and extension of the thumb IPJ. His key pinch strength at two years and two months postoperatively was 11 kg (versus 8 kg on the contralateral side).

## Discussion

The incidence of flexor tendon injuries is quoted as 4.83 per 100,000 persons [3]. While tendons may successfully be repaired, re-rupture and postoperative adhesions frequently complicate clinical outcomes.

Tendons consist of endotenon, paratenon, and epitenon. Intrasynovial tendons are also enveloped in synovial covering and sheath. This sheath provides the tendon with the environment for a maximal excursion, which relies on the tendon gliding within the sheath. In cases of injury and repair, this gliding motion may be inhibited by cicatrix and adhesions that form during the healing process. As such, interventions are sought to optimize tendon gliding after repair to improve the resulting range of motion and function.

Many studies have examined the use of biologic agents as an adjunct to achieve this goal. Hyaluronic acid is one such treatment that has emerged. Sun et al. [4] demonstrated decreased resistance to tendon gliding in their canine model. Other studies have found similar results with these adjuncts [5,6].

The use of an HA/alginate sheet in the presented case demonstrated a lack of adhesion formation over the applied area on inspection at the second procedure. This case provided the rare opportunity to visualize a tendon repair after the patient had completely healed and been cleared for full activity. Figure 2 displays the tendon at the second operative intervention for the patient’s unrelated surgery. The tendon repair was intact with no adhesion formation in the area where the HA wrap had been placed, while proximal to this area, adhesions were noted.

It is crucial to highlight that this repair and the use of the HA/alginate sheet were in conjunction with early postoperative rehabilitation. Current rehabilitation protocols after tendon repair aim to improve clinical outcomes by maximizing tendon excursion, employing an active and passive range of motion. One such protocol, which was used in this case, is the Duran protocol, which has been shown to decrease the risk of re-rupture while having a slightly higher risk of decreased postoperative digit ROM [7]. In this case, the protocol was selected given the frayed nature of the tendon ends that were repaired. The presented patient was prescribed occupational therapy at the first postoperative visit and was fitted for a splint to protect the repair. His progress was monitored over several visits, and he continued to improve through the guidance of occupational therapy. At his final visit, the patient was satisfied with his result and demonstrated no limitations.

## Conclusions

Tendon injuries pose a unique challenge to treating surgeons with the ultimate goal of providing the patient with a strong tendon repair without adhesions to allow for maximum postoperative function. This case highlights that biologic adjuncts such as a HA/alginate sheet, when used in conjunction with proper tendon repair and a postoperative rehabilitation protocol, can minimize adhesion formation, prevent unwanted postoperative tethering, and lead to successful clinical outcomes.
